# Rheumatologic and autoimmune features of inborn errors of immunity: Implications for diagnosis and management

**DOI:** 10.70962/jhi.20250034

**Published:** 2025-07-23

**Authors:** Joshua M. Tobin, Megan A. Cooper

**Affiliations:** 1 Department of Pediatrics, Division of Rheumatology/Immunology, Washington University in St. Louis, St. Louis, MO, USA

## Abstract

Inborn errors of immunity (IEI) are monogenic disorders of the immune system that frequently present with autoimmunity or autoinflammation, necessitating multispecialty care. In many cases, patients may present for rheumatologic evaluation prior to a genetic diagnosis, highlighting the need for recognition of an underlying IEI with immune dysregulation. Here, we review IEI that can present with rheumatologic and autoimmune complications and the role of genetic testing for establishing a molecular diagnosis and devising personalized treatment plans to improve patient outcomes.

## Introduction

Inborn errors of immunity (IEI) are a broad class of monogenic diseases that affect immune system function. The initial discovery of IEI in the 1950s–1960s were made in patients with infectious susceptibility and primary immune deficiencies; however, it is now well recognized that the clinical spectrum of IEI encompasses autoimmunity, autoinflammation, bone marrow failure, lymphoproliferation, severe atopy, and/or malignancy. Several large cohort studies have identified autoimmunity, immune dysregulation, or autoinflammation in approximately one third of patients with IEI (1, 2, 3), and inflammatory manifestations were the initial presentation in 18% of one cohort (4).

IEI presenting with symptoms that overlap with more common systemic autoimmune conditions represent a significant clinical challenge; however, early identification and treatment are associated with improved patient outcomes and frequently require a multidisciplinary approach. Clinical challenges include identification, diagnosis, and immune-modulating therapy for patients with rheumatologic disease associated with an IEI. Here, we review the IEIs that present with rheumatologic manifestations, discuss the clinical approach to identifying IEIs, interpretation of genetic testing, and treatments targeted toward IEI.

## IEI associated with rheumatologic disease

**Figure 1.**
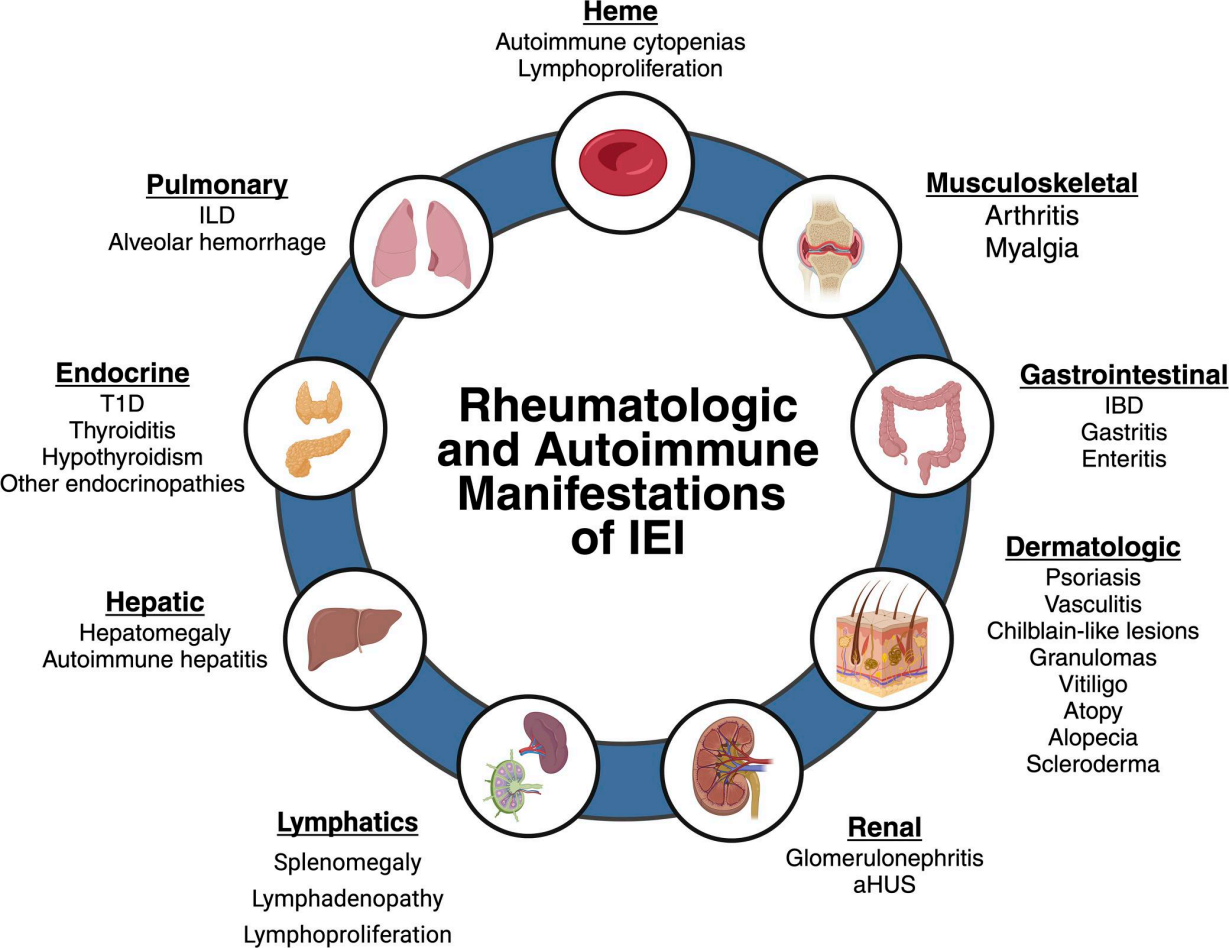
**Rheumatologic and autoimmune manifestations of IEI.** ILD, interstitial lung disease. Created in BioRender: Tobin, J. (2025), https://BioRender.com/f82e845.

There are more than 500 IEI, most of which are genetically defined and classified by the International Union of Immunological Societies (IUIS) based on mechanism and associated disease (5). IEI can have a wide range of autoinflammatory, rheumatologic, and autoimmune presentations affecting nearly every organ system, broadly referred to as immune dysregulation (Fig. 1). Rheumatologists treat systemic inflammatory conditions including autoimmunity and autoinflammation causing symptoms such as fevers, rashes, musculoskeletal disorders, vasculitis, autoimmune cytopenias, and end organ dysfunction (e.g., lung and kidney disease) (2). Thus, pediatric and adult rheumatologists are potentially one of the first specialists to see patients with IEI complicated by these conditions. For example, patients with NLRC4-associated autoinflammatory syndrome presenting with joint pain, fevers, and rashes may be evaluated by rheumatologists for systemic lupus erythematosus (SLE) or systemic juvenile arthritis. Patients with CTLA4 haploinsufficiency may present to a rheumatologist with joint pain and interstitial lung disease but also have significant antibody deficiency that is important to recognize. Rheumatologists are also often part of multispecialty care teams that treat other autoimmune and autoinflammatory conditions that may initially present other specialists; for example, patients with STAT3 gain-of-function (GOF) syndrome with early onset type I diabetes (T1D), arthritis, and autoimmune cytopenias and lymphoproliferation may present to endocrinology, rheumatology, and/or hematology. Here, we highlight presentations of IEI relevant for rheumatologists to recognize.

IEI can be broadly classified as those that have significant susceptibility to infection versus IEI with predominantly immune dysregulation, including rheumatologic manifestations, Although there is overlap between the two, as patients with IEI that primarily present with infection may later develop immune dysregulation and those with immune dysregulation may be at increased risk for infection, these categories can be useful as a framework for clinicians considering therapeutic approaches.

### IEI with significant susceptibility to infection

Among the earliest documented cases of rheumatologic presentations in immunodeficiencies were the discoveries of hyper-IgM (HIGM) syndrome in 1961 (6, 7) and Omenn syndrome in 1965 (8). However, we now understand that rheumatologic and autoimmune manifestations are widespread in IEI with infectious susceptibility (Table 1).

**Table 1.** IEI with predominant susceptibility to infection

Classification	Clinical syndrome	Rheumatologic/autoimmune manifestations	Examples of genes identified
SCID	Omenn syndrome	Lymphadenopathy, hepatosplenomegaly, increased IgE, and erythroderma	*RAG1*, *ADA*, *RMRP*, *DLCRE1C*, *LIG4*, *IL2RG*, and *IL7RA*
	GVHD of transplacental maternal engraftment	Morbilliform erythema, papular dermatitis, and erythroderma	SCID-associated genes
CID	CID-G/AI	Granulomas of the skin, mucosa, lungs, and adenoids ANCA vasculitis Autoimmune cytopenias Myasthenia gravis	*RAG1* and *RAG2*
	HIGM syndrome	Increased circulating autoantibodies Increased risk of diabetes mellitus and autoimmune cytopenias	*CD40L*, *CD40*, *AICDA*, *UNG*, *MSH6*, *CTNNBL1*, and *APRIL*
	APDS	Lymphadenopathy, lymphoproliferation, and autoimmunity (cytopenias and glomerulonephritis)	*PIK3CD* and *PIK3R1*
	Disorders of T cell activation	Inflammatory arthritis and IBD	*ZAP70*, *SLP76*, and *LAT*
	Deficiencies in thymic development	Omenn syndrome findings (*FOXN1*)	*FOXN1* and *TBX1*
	Chr22q11.2 microdeletion syndrome	Increased likelihood of ITP and JIA	Chr22q11.2
	WAS	AIHA, vasculitis, and glomerulonephritis	*WASP*
Predominantly antibody deficiency	CVID	SLE, IBD, chronic lung disease, and liver disease (hepatitis C, granulomas, and idiopathic liver disease)	*TNFRSF13B* (TACI), *TNFRSF13C* (BAFFR), *CD19*, *CD21*, *CD81*, *NFKB1*, *NFKB2*, and *PTEN*
	Agammaglobulinemia	IBD, rheumatoid arthritis, fatigue, chronic diarrhea, rash, and joint pain	*BTK* and *SPI1*

A summary of the major IEI that present with predominant susceptibility to infections, categorized into SCID, CID, CID with syndromic features, and predominantly antibody deficiencies. ANCA, antineutrophil cytoplasmic antibody; WASP, WAS protein.

#### Severe combined immunodeficiency (SCID)

SCID is a group of IEI defined by a defect in the hematopoietic compartment leading to absent or severely impaired T cell development and function, often with profound defects in B cells and/or natural killer (NK) cells (9). While most patients have typical SCID with a near complete loss of T cells, ∼1 out of every 4 SCID patients have “leaky” or atypical SCID due to hypomorphic variants in SCID-associated genes, with *RAG1*, *ADA*, and *RMRP* accounting for 57% of these cases (10). Criteria for leaky/atypical SCID include: low T cell count for age (with <0.6 × 10^3^/μl qualifying at any age), evidence of an oligoclonal T cell population, and/or either abnormal T cell receptor excision circles (TRECs) or a low proportion of naïve T cells (<20% of CD4^+^ T Cells) (9). A subset of leaky/atypical SCID cases present with Omenn syndrome, with classic features of enlarged lymph nodes, hepatosplenomegaly, elevated IgE, and erythroderma, due to autoreactive T cells escaping central tolerance mechanisms in the thymus with expansion in the periphery (11, 12). A similar constellation of symptoms can be caused by transplacental maternal engraftment of T cells in patients with typical SCID due to a failure to reject maternal T cells (13, 14), leading to a graft-vs-host disease (GVHD)–like phenotype, often including liver and gastrointestinal tract involvement, eosinophilia, and thrombocytopenia (15).

Newborn screening of TREC has greatly improved the detection and early treatment of SCID; however, rare SCID cases can be missed by newborn TREC screening, with most of these patients having leaky/atypical SCID (16, 17, 18). Therefore, consideration of the range of SCID presentations and testing even with a normal TREC screen is important.

#### Combined immunodeficiencies (CID)

CID affect the function of both B and T cell compartments, generally less severely than SCID, and many CIDs have features of immune dysregulation. Relevant to rheumatologists, hypomorphic variants in SCID-associated genes can cause phenotypes of CID with autoimmunity. Hypomorphic *RAG* variants can allow partially preserved recombinase activity (∼5–30%) and result in a later presentation of CID with granuloma or autoimmunity (CID-G/AI) (19). CID-G/AI was initially reported among three unrelated girls with granulomatous disease in the skin, mucous membranes, adenoids, and lungs, severe complications due to viral infection (20). Additional patients with CID-G/AI have been identified presenting with autoimmune conditions, including vasculitis, autoimmune cytopenias, and myasthenia gravis (21, 22). Many patients have circulating autoantibodies, including neutralizing antibodies against IFN-α and IFN-ω (23). Hypomorphic *RAG2* variants have also been associated a HIGM-like phenotype with expanded autoreactive B cells (24, 25), with the same variant resulting in multiple phenotypes (26).

While loss of T cell function leads to infectious susceptibility, residual function of the affected gene may cause immune dysregulation and autoimmunity. Although patients with *ZAP70* deficiency generally have a SCID-like phenotype, 20% present with autoimmunity, most commonly ulcerative colitis and/or autoimmune cytopenias (27). In one case, siblings with both hypomorphic and hypermorphic variants in *ZAP70* developed a predominant autoimmune phenotype (28). Similar phenomena have also been seen with *LAT* and *SLP76* hypomorphic variants (29, 30). Pathogenic variants in *PTCRA*, which encodes pre-TCRa, have recently been reported (31). Four out of 10 patients developed lymphoproliferation, infections, or autoimmunity in their teenage years or early adulthood, while six were clinically asymptomatic.

Classic HIGM, caused by CD40L deficiency (X linked) or, rarely, CD40 deficiency (autosomal recessive) (32), presents with susceptibility to opportunistic infections due to the inability to provide CD40 signaling to dendritic cells and macrophages, resulting in subsequent deficiencies of IL-12 production to induce T cell activation and polarization. (33, 34). Patients with HIGM also have an increased risk of autoimmunity, including T1D, thrombocytopenia, and autoimmune hemolytic anemia (AIHA) (35). Patients with HIGM due to deficiencies in class switch recombination genes, such as *AICDA* and *UNG*, also have autoimmunity but have a phenotype more restricted to B cell dysfunction without opportunistic infections (36). Increased titers of circulating autoantibodies in patients with HIGM suggest that a loss of B cell tolerance plays a role in the development of autoimmunity (37).

Activated PI3K delta syndrome (APDS) is due to autosomal dominant GOF variants in *PIK3CD* (APDS1) or loss-of-function (LOF) variants in the PI3K regulator *PIK3R1* (APDS2). Patients with APDS often have a phenotype resembling common variable immunodeficiency (CVID) with reduced naïve B cells and impaired antibody production, but also have significant lymphadenopathy, lymphoproliferation, and autoimmunity. Among 53 individuals with APDS, >30% of patients presented with autoimmunity, most commonly autoimmune cytopenias; however, other presentations of glomerulonephritis and thyroid disease were also identified (38).

A subset of CID also affects non-immunologic compartments, resulting in associated syndromic features. Wiskott–Aldrich syndrome (WAS) is caused by variants in the WAS protein, which affects actin polymerization (39). Patients often present in infancy with hemorrhage and/or petechiae and eczema (40). Autoimmunity is present in ∼40% of patients, including AIHA, vasculitis, and renal disease (41). *FOXN1* deficiency affects thymic stromal cell development. Patients have incomplete or absent T cell development, with one characteristic feature also including nail dystrophy (42). Approximately 50% of patients present with symptoms resembling Omenn syndrome (43). Heterozygous LOF variants in *FOXN1* in children can result in less severe susceptibility to infection, low levels of TRECs, and T cell lymphopenia, and one patient was reported with severe thrombocytopenia (44). Adults with the same variant had persistent CD8 lymphopenia but improved CD4 counts and reduced susceptibility to infection, suggesting *FOXN1* gene dosage is important early in life for CD8 development. Chr22q11.2 microdeletion syndrome, also known as DiGeorge syndrome, is caused by a deletion in 22q11.2, with >90% of cases arising de novo (45). Patients typically present with congenital abnormalities such as cardiac defects, cleft palate, and low-set ears (46). Most have some degree of thymic hypoplasia due primarily to haploinsufficiency of *TBX1* in the deletion (47). Immunologic findings include low or absent T cells and susceptibility to infection with a significantly increased risk of autoimmunity, as ∼8% of patients present with autoimmune manifestations (48), most commonly immune thrombocytopenic purpura (ITP) (4% prevalence) and juvenile idiopathic arthritis (JIA) (2% prevalence compared with 0.1% in the general population) (49).

Immune dysregulation is also associated with more common syndromic diseases. In particular, patients with trisomy 21 (Down syndrome) have high rates of autoimmunity, including hypothyroidism in ∼1/3 of patients, an increased risk (approximately fourfold) of developing T1D with early onset, and celiac antibodies in 10% of individuals (50, 51, 52). Patients have elevated levels of multiple cytokines, expanded atypical B cells, and abnormal thymic architecture. (53, 54, 55). Additionally, patients generally have elevated type I IFN signaling, in part due to the presence of *IFNAR1* and *IFNAR2* on chromosome 21, and have hyperinflammatory responses to SARS-CoV-2 and other viral infections (56). JAK inhibitors (JAKinibs) have been proposed as a therapy for patients with trisomy 21, similar to those with type I interferonopathies (57).

#### Predominantly antibody deficiencies

Predominantly antibody deficiencies encompass a wide range of B cell deficiencies, ranging from total lack of circulating immunoglobulins to functional defects despite normal circulating immunoglobulin levels. A clinical phenotype of CVID, defined by recurrent infections with low levels of immunoglobulin and poor vaccination responses, is the most common form of antibody deficiency. In most cases, a genetic etiology of CVID is not identified; however, there are several monogenic IEI with CVID phenotypes, including genes encoding for the BCR complex (CD19, CD21, and CD81), NFκB-associated defects, or the BAFF receptors (TACI and BAFF-R), which confer an increased risk of CVID. A longitudinal study of CVID patients over 40 years found that 68% of patients have at least one inflammatory or autoimmune complication, with ITP, AIHA, and Evans syndrome being the most common and reports of other autoimmune disorders such as SLE, inflammatory bowel disorder (IBD), lung disease, psoriasis, and/or liver autoimmunity (58). Notably, nearly 30% of CVID patients develop chronic lung disease, and ∼11% develop bronchiectasis (58, 59). In a cohort of CVID patients with ITP, 9 out of 15 patients presented with ITP an average of 4.5 years prior to their CVID diagnosis (60). Therefore, CVID patients may be initially referred to a wide range of clinical specialties, and screening for antibody levels and responses should be considered in patients with autoimmunity, particularly with recurrent infections and/or when disease does not respond to treatment as expected.

Autoimmunity and autoinflammation are also seen in other antibody deficiencies. Patients with X-linked agammaglobulinemia (XLA) due to *BTK* deficiency have an increased incidence of autoimmunity despite no circulating antibodies. Up to 35% of patients with XLA have gastrointestinal manifestations, and 10% have diagnoses of IBD or enteritis (61). About half of patients (45%) experience arthritis, which often presents prior to their diagnosis of XLA (62). Most cases of arthritis in XLA are thought to be of infectious etiology; however, case reports have identified patients with rheumatoid arthritis and renal disease (63, 64). Other forms of agammaglobulinemia may also have increased risk of autoimmunity. Among 25 individuals with LOF variants of *SPI1*, 8 patients had IBD, 2 had JIA, and 2 had T1D, suggesting a significantly higher prevalence than the general population (65).

### IEI in which rheumatologic disease is a primary clinical feature

In some cases, rheumatologic features are the primary clinical presentation of an IEI, and affected patients are likely to present to and be treated by a rheumatologist prior to a genetic diagnosis. IEI with prominent rheumatologic disease can broadly be classified into disorders of immune regulation, innate immune defects, and autoinflammatory conditions (Table 2). Nearly half of the IEI new to the 2024 IUIS report can include a rheumatologic presentation (5), highlighting the necessity for rheumatologists to maintain a high index of suspicion for IEI in their patients.

**Table 2.** IEI with predominant rheumatologic manifestations

Classification	Clinical syndrome	Rheumatologic/autoimmune manifestations	Examples of genes identified
Disorders of immune dysregulation	APECED	Chronic mucocutaneous candidiasis, hypoparathyroidism, primary adrenal insufficiency, diabetes mellitus, and JIA	*AIRE*
	ALPS	Lymphoproliferation, lymphadenopathy, splenomegaly, and autoimmune cytopenias	*FAS* and *FADD*
	Tregopathies	IPEX (T1D, autoimmune enteropathy, eczema, dermatitis, alopecia, and psoriasis-like lesions)	*FOXP3*, *CD25*, *STAT5B*, *LRBA*, *CTLA4*, and *DEF6*
		Multi-organ lymphocytic infiltration	
		T1D, hypoparathyroidism, and JIA	
	Disorders of cytokine signaling	T1D, cytopenias, SLE, lymphadenopathy, and multi-organ autoimmunity	*STAT1*, *STAT3*, *STAT4*, *STAT6*, *JAK1*, *SOCS1*, *PTPN2*, *ISG15*, and *USP18*
	HLH	Fever, cytopenias, elevated ferritin, hypertriglyceridemia, and encephalitis	*PRF1*, *STX11*, *STXBP2*, and *UNC13D*
Innate immune defects	Monogenic lupus	SLE	Complement genes, *DNASE1*, *DNASE1L3*, *TLR7*, and *UNC93B1*
	Disorders of complement regulators	Vasculopathies and aHUS	*CFH* (factor H), *CFI* (factor I), *CD45* (membrane cofactor protein), and autoantibodies targeting factor H
Autoinflammatory disorders	Interferonopathies	Severe neurologic findings	AGS (*TREX1*, *RNAASEH2A*, *RNASEH2B*, *RNASEH2C*, *TREX1*, *ADAR*, and *IFIH1*), *COPA*, *STING1*, and *ADA2*
		Skin manifestations (chilblain-like lesions)	
		Interstitial lung disease	
	Inflammasomopathies	Periodic fevers, peritonitis, synovitis, and pleuritis	*MEFV* (FMF), *NLRP3*, *PLCG2*, and *NLRC4*
	Non-inflammasomopathy inflammatory disorders	Oral and genital ulcers, arthritis, erythema nodosum, recurrent fevers, and very early onset IBD	*TNFRSF1A* (TRAPS) *TNFAIP3* (A20), *RELA*, and *IKBKG*
	VEXAS syndrome	Alveolitis, chondritis, thromboembolisms, dermatoses, and cutaneous vasculitis	*UBA1* (somatic)

A summary of the major IEI that present with rheumatologic, autoimmune, and autoinflammatory conditions as their primary manifestations. FMF, familial Mediterranean fever; TRAPS, tumor necrosis factor receptor-associated periodic syndrome.

#### Disorders of immune regulation

**Figure 2.**
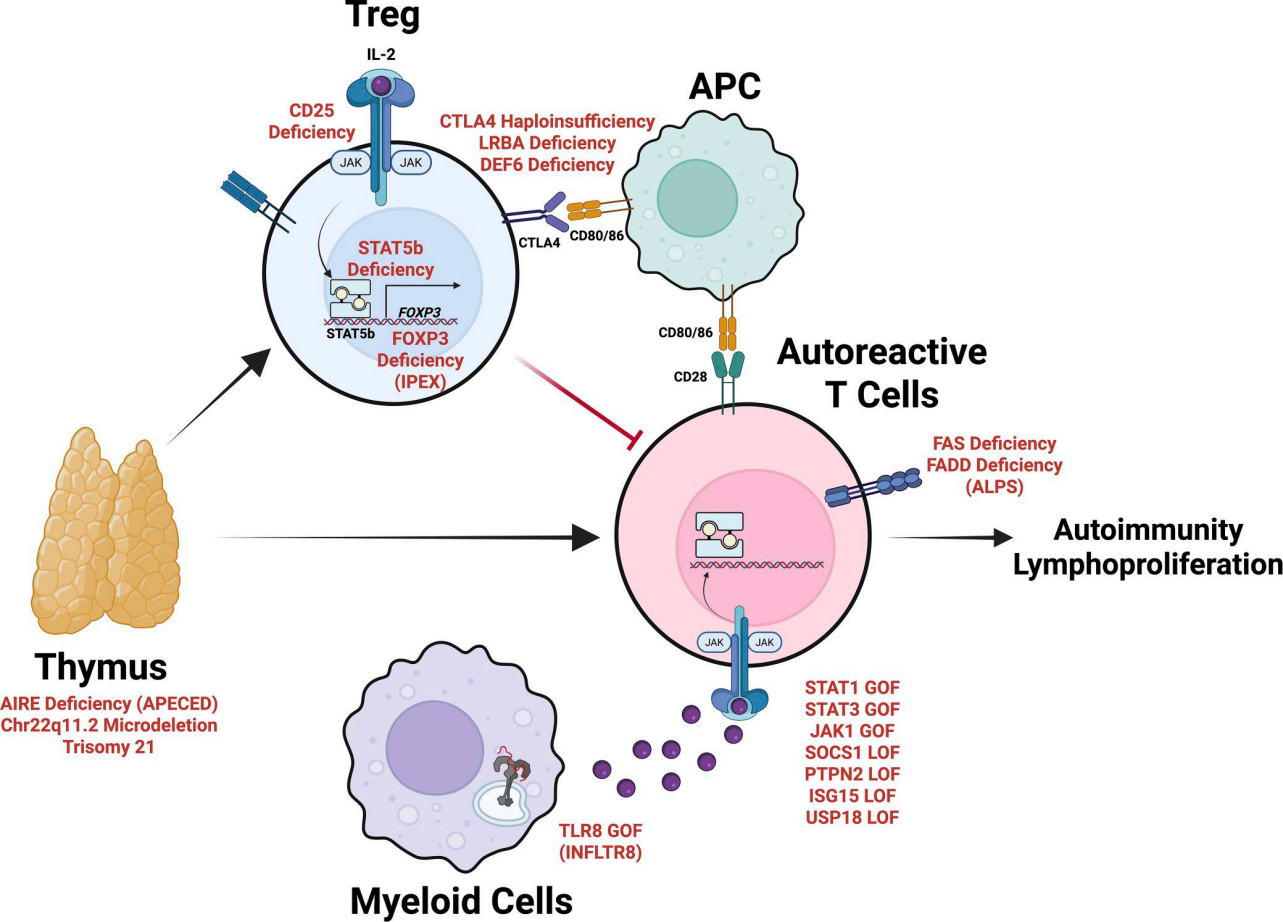
**Mechanisms of T cell dysfunction leading to immune dysregulation.** A summary of representative IEI and genetic mechanisms of T cell dysfunction leading to immune dysregulation. Created in BioRender. Tobin, J. (2025) https://BioRender.com/o30j099.

Immune dysregulation disorders represent a group of IEI primarily characterized by breakdown of immune tolerance and clinical features with prominent autoimmunity. The initial recognition of these primary immune regulatory disorders (PIRD) were monogenic IEI, leading directly to impaired T cell tolerance, including those due to defects in *AIRE*, *FAS*, and *FOXP3*, and this group of disorders has expanded significantly over the last several decades, including disorders of cytokine signaling (66). Many PIRD induce dysregulation of T cells that leads to lymphoproliferation, autoinflammation, and autoimmunity. T cell dysregulation can occur at multiple stages of T cell development in both T cell intrinsic and extrinsic manners, including inappropriate thymic selection, regulatory T cell (Treg) defects, and cytokine signaling abnormalities (Fig. 2).

Autoimmune polyendocrinopathy candidiasis ectodermal dystrophy (APECED) was one of the first documented disorders of immune regulation. Originally characterized in the 1960s by scientists studying adrenal insufficiency, APECED is an autosomal recessive condition caused by LOF variants in *AIRE*, which encodes the transcription factor AIRE that is a core component of central T cell tolerance (67, 68, 69). APECED results in autoreactive T cells, as the lack of AIRE restricts the self-antigens expressed by thymic epithelial cells during selection (70). Patients with APECED were originally described to have a triad of chronic mucocutaneous candidiasis, hypoparathyroidism, and primary adrenal insufficiency (71); however, expanded diagnosis and recognition of APECED has revealed multisystem autoimmunity, also including autoimmune gastritis, hepatitis, and pneumonitis, vitiligo, and Sjogren’s-like syndrome (72). While less common, JIA has also been described (73). Interestingly, patients with APECED have susceptibility to mucocutaneous candidiasis and severe viral infection, including COVID-19, due to the presence of autoantibodies to relevant cytokines including Th17-associated cytokines and type I IFNs, respectively (74, 75).

Autoimmune lymphoproliferative syndrome (ALPS) is due to autosomal dominant and somatic defects in the genes encoding FAS or FADD, leading to accumulation of activated T cells that have presumably escaped peripheral tolerance mechanisms. Patients present with lymphoproliferation and autoimmune cytopenias with increased risk of malignancy (76, 77). Approximately 14% of patients with ALPS may experience other autoimmunity, including glomerulonephritis and hepatitis (78).

Immune dysregulation, polyendocrinopathy, enteropathy, X-linked syndrome (IPEX) is caused by variants in *FOXP3*, encoding the lineage-defining transcription factor of Tregs. The lack of functional Tregs results in autoreactive T effector cells and multi-organ autoimmunity. Affecting males, IPEX often presents with broad multi-organ autoimmunity, commonly autoimmune enteropathy, T1D, and dermatologic manifestations of eczema, dermatitis, psoriasis-like lesions, and alopecia (79). Cytopenias and membranous nephropathy are also seen in some patients with IPEX (80). Other examples of genes leading to PIRD characterized by predominant Treg dysfunction include deficiencies of *CD25* and *STAT5B*, both required for Treg differentiation, and haploinsufficiency of *CLTA4* or deficiency of *LRBA* and *DEF6*, which encode proteins needed for cell-surface expression CTLA4 and Treg function (81, 82, 83). Of particular relevance to rheumatologic disease, CTLA4 is highly expressed by Tregs and functions as an immune checkpoint to inhibit activated T cell responses (84). Autosomal dominant CTLA4 haploinsufficiency results in lymphocytic infiltration and disease of multiple organs, including the arthritis, intestinal, pulmonary, and renal manifestations (85, 86). LRBA is required for vesicular trafficking of CTLA4 and thus required for its cell-surface expression, resulting in similar phenotype as CTLA4 haploinsufficiency, although with more prominent lung disease (87). Diagnosis of CTLA4- or LRBA-associated disease informs therapy given the availability of CTLA4-Ig. Deficiency of PD-1, another checkpoint inhibitor, was identified in a patient presenting with T1D, hypothyroidism, JIA, and susceptibility to tuberculosis infection. Although aggressive antimicrobial therapy allowed the patient to recover from abdominal tuberculosis infection, he later developed autoantibodies against collagen α3 and progressed to fatal respiratory failure due to alveolar hemorrhage (88).

Immune dysregulation can also occur due to perturbations of cytokine signaling. The JAK/STAT pathway plays a critical role in propagating signals from multiple cytokines. IEI have been associated with most of the STAT genes (89). STAT1 is downstream of type I and II interferon signaling, and STAT1 GOF syndrome was initially discovered as associated with Mendelian susceptibility to mycobacterial disease; however, a large cohort of 274 patients demonstrated a high rate of autoimmunity in ∼37% of patients, including T1D, hypothyroidism, cytopenias, and SLE (90). STAT3 GOF syndrome was initially reported in a cohort with T1D and in children with lymphadenopathy, cytopenias, and multi-organ autoimmunity (91, 92, 93, 94). Additional studies in the decade since its discovery have elucidated a wide clinical spectrum of presentations, and cohort study of 191 patients with *STAT3* GOF identified lymphoproliferative disease as the most common manifestation (73%), followed by cytopenias (67%) (95). STAT4 GOF was identified in four patients from three families who presented in childhood with pansclerotic morphea, including mucosal ulcerations and skin sclerosis, with some patients having additional joint swelling and contractures (96).

JAK1 GOF results in immune dysregulation with atopic dermatitis, autoimmune thyroid disease, and eosinophilia and is associated with dominant germline or mosaic variants (97, 98). In a large study of JAK1 GOF, the clinical phenotypes of 59 individuals with germline variants suggests an emerging pattern of a syndromic phenotype atopy, colitis, and autoimmunity that responds to JAKinibis (99).

LOF or haploinsufficiency of regulators of STAT signaling can lead to enhanced JAK/STAT signaling and similar disease phenotypes as JAK/STAT GOF disorders. *SOCS1* encodes a negative regulator of STAT signaling, and patients with SOCS1 haploinsufficiency present with autoimmune cytopenias, SLE, and polyarthritis, and other autoimmune manifestations (100). Of 61 patients with SOCS1 haploinsufficiency, 37% presented with rheumatologic manifestations, including SLE, Sjogren’s syndrome, and rheumatoid arthritis. Additionally, many patients presented with other autoimmune manifestations, including autoimmune cytopenias (39%), lymphoproliferation (37%), and inflammatory gastrointestinal manifestation (36%), (101). *PTPN2* encodes a phosphatase that negatively regulates JAK/STAT signaling. Pathogenic LOF variants in *PTPN2* were initially described in patients with autoimmune enteropathy and CID, and more recently in six patients with SLE and autoimmune cytopenias (102, 103, 104, 105).

We identified somatic and germline GOF variants in *TLR8* presenting with inflammation, neutropenia, bone marrow failure, lymphoproliferation, caused by TLR8 (INFLTR8), which has additional presentations of antibody deficiency, CD8 T cell dysregulation, and in some cases large granular lymphocytic leukemia (106). *TLR8* is expressed exclusively in myeloid cells, and in mosaic patients, GOF variants are expressed in <20% of cells. These patients have high levels of systemic inflammatory cytokines and significant T cell dysfunction with reversal of CD4/CD8 ratios and accumulation of antigen-experienced CD8 T cells that together with the mosaic nature of most TLR8 GOF variants suggests a cell-extrinsic effect of myeloid cells expressing mutant *TLR8* on the adaptive immune response.

Rheumatologists diagnose and manage macrophage activation (MAS) syndrome in the context of systemic JIA and other rheumatologic diseases. There is significant overlap between MAS and hemophagocytic lymphohistiocytosis (HLH), either primary due to a monogenic IEI or secondary to infection or other forms of uncontrolled inflammation, and particularly in young children the differential diagnosis between MAS, secondary HLH, and primary HLH can be challenging. Primary HLH are a group of IEI categorized as immune dysregulation and associated with defects in genes required for T and NK cell cytotoxicity, leading to an inability to regulate the immune response to infection; for example, *PRF1*, *STX11*, *STXBP2*, and *UNC13D* (107). Secondary HLH can be seen in many IEI, likely due to a combination of inability to control infection and a dysregulated immune response, and may present similarly to MAS in systemic rheumatologic conditions (108). Some examples of IEI that can lead to secondary HLH include STAT1 GOF and LOF, *STAT2* deficiency, *CYBB* deficiency (the cause of XL CGD), IFNAR1 and IFNAR2 deficiencies, and GATA2 deficiency (109, 110, 111, 112, 113, 114).

#### Innate immune defects and complement disorders

The innate immune system plays a critical role in sensing pathogens or damage and providing a first-line response to injury. While many IEI affecting the innate immune system cause susceptibility to infection, aberrant sensing or responses of the innate immune system can also result in significant inflammation and autoimmunity. The complement system is critical not only for clearing pathogens but also for the removal of apoptotic cells and immune complexes. Deficiencies in early components of the classical complement pathway, including C1q, C1r, C1s, and C4, are associated with monogenic lupus in almost all patients (115, 116). C2 is the most common complement deficiency (117) but has a weaker association to SLE, with an estimated 10–30% of patients developing SLE. Genes involved in the nucleic acid degradation pathway, such as *DNASE1* and *DNASE1L3*, can also cause monogenic lupus, likely due to the inability to clear DNA from apoptotic debris (118, 119, 120). Lastly, GOF variants in *TLR7*, along with *UNC93B1* encoding a chaperone protein associated with TLR7, are associated with monogenic SLE (121, 122, 123).

LOF variants in regulators of complement, such as *CD46*, *CFH* (factor H), and *CFI* (factor I), can result in hyperactivation of the complement pathway with increased consumption of C3, and patients frequently present with atypical hemolytic uremic syndrome (aHUS) (124, 125). Factor I deficiency may also manifest with vasculitis and CNS inflammation, and the C5 inhibitor, eculizumab, was effective in treating these patients (126, 127).

#### Autoinflammatory disorders

Autoinflammatory disorders occur due to antigen-independent activation of the immune system. The characterization of Aicardi–Goutières syndrome (AGS) and its association with type I IFN signaling led to the discovery and categorization of type I interferonopathies (128, 129). Patients often present with severe neurologic findings, and ∼40% have autoinflammatory skin manifestations consisting of chilblain-like lesions on the extremities. While many type 1 interferonopathies present similarly to AGS, there is heterogeneity in their presentations (130). For example, COPA syndrome, arising from variants in *COPA* that encodes an ER transport protein, and STING-associated vasculopathy with onset in infancy (SAVI) both present with interstitial lung disease, but patients with COPA syndrome often have alveolar hemorrhage, which is only seen in a handful of patients with SAVI syndrome (131, 132, 133). Furthermore, patients with COPA are more likely to experience arthritis and have renal involvement, whereas patients with SAVI syndrome are more likely to have skin vasculopathy (134).

Deficiency of ADA2 (DADA2) has a variable clinical presentation that in some patients presents with either a vasculopathy phenotype resembling polyarteritis nodosum with early onset stroke or pure red cell aplasia and bone marrow failure, with some patients also having immune deficiency and a CVID-like phenotype (135, 136). DADA2 is relatively common compared to other monogenic IEI, with an estimated 30,000 cases worldwide (137) and should be considered in patients of all ages presenting with a consistent phenotype (138). Relevant to rheumatologists, an ADA2 functional test can provide rapid results to guide clinical management, and TNF inhibitors have shown significant success in treating vasculopathy in DADA2 (139).

Inflammasomopathies include monogenic diseases causing periodic fever syndromes. Familial Mediterranean fever is caused by variants in the *MEFV* gene, which encode the protein pyrin that is important for inflammasome assembly. Patients have recurrent fevers and can develop spontaneous painful inflammatory episodes in multiple organ systems, including peritonitis, synovitis, and pleuritis (140), and if untreated, can develop amyloidosis and end-organ damage (141). Patients experience periodic episodes of fever that can be associated with inflammation in multiple organ systems, including myalgia, abdominal pain, and lymphadenopathy, and respond to IL-1 inhibition (142).

Cryopyrin-associated periodic syndrome encompasses a spectrum of phenotypes caused by GOF variants in *NLRP3* that often present in the neonatal period and can range from severe inflammation with fevers and arthropathy to recurrent episodes of urticaria-like rashes, sometimes accompanied by fever (143). PLCγ2 signaling has broad effects across immune cells, and hypermorphic variants in *PLCG2* cause autoinflammation and PLCγ2-associated antibody deficiency and immune dysregulation (144), thought to be at least partially due to hyperactivation of the NLRP3 inflammasome (145). NLRC4 GOF results in a phenotype distinct from other inflammasomopathies with fever, elevated ferritin and triglycerides, and pancytopenia resembling HLH that is responsive to anti-IL-18 therapy (146, 147).

Rheumatologists also treat other non-inflammasomopathies causing periodic fevers, such as tumor necrosis factor receptor-associated periodic syndrome due to variants in *TNFRSF1A* (148), as well as those affecting NF-κB pathways leading to autoinflammation. Haploinsufficiency of A20 (HA20), encoded by *TNFAIP3*, can manifest with childhood-onset Behçet-like disease with additional recurrent fever, lymphadenopathy, skin manifestations, and autoimmune cytopenias (149). A20 is a ubiquitin-editing enzyme that acts as a negative regulator of NF-κB and other pathways, including the NLRP3 inflammasome. Loss of A20 function results in increased TNF and IL-1β, and therapies targeting TNF and IL-1β have been successfully used, as well as JAKinibs due to the high type 1 interferon signature (150). Haploinsufficiency of p65, encoded by *RELA*, can present in patients with early onset mucosal ulcers, recurrent fevers, and leukocytosis (151, 152). Patients with dominant negative variants in *RELA* have additional inflammatory presentations compared with RELA haploinsufficiency, including IBD, JIA, and skin manifestations, resembling type 1 interferonopathies (153). *IKBKG* encodes NEMO, an adaptor protein essential for NF-κB signaling downstream of RIG-I and TLR3. While patients with NEMO deficiency present with a CID, alternative splicing variants leading to overall increased NF-κB activity and autoinflammatory syndrome known as NEMO-deleted exon 5 autoinflammatory syndrome (NDAS) (154, 155). NDAS presents with recurrent fever, hepatosplenomegaly, ITP, hypogammaglobulinemia, and nodular skin rashes, with TNF inhibitors demonstrating success in treating disease (155).

Somatic variants in the gene encoding the ubiquitin-activating enzyme UBA1 cause an adult-onset multisystem autoinflammatory disorder, known as vacuoles, E1 enzyme, X-linked, autoinflammatory, somatic (VEXAS) syndrome (156). Since its original description in 2020, the clinical spectrum of VEXAS has expanded considerably and includes recurrent fevers, alveolitis, arthritis, chondritis, and thromboembolisms, as well as skin manifestations, such as dermatoses and cutaneous vasculitis (157). Myelodysplastic syndrome and other hematologic disease are also prominent in patients with VEXAS syndrome and can come before or after autoinflammatory disease. Although many IEI present in early childhood, VEXAS syndrome highlights the importance of considering the presence of IEI in adult patients too and the need to consider somatic mosaicism as a cause of disease in patients with immune dysregulation.

## Diagnosis of IEI in the rheumatology clinic

### Genetic testing for IEI

IEI present with a vast clinical spectrum that encompasses nearly every form of autoimmunity and immune dysregulation, and many patients may initially present to rheumatology or other clinical specialties prior to having a genetic diagnosis. In some cases, treatment of rheumatic conditions with immune suppression or modulation may reveal an IEI; for example, poor B cell reconstitution and hypogammaglobulinemia after receiving B cell depletion therapies (158).

The identification of a genetic diagnosis can alter clinical diagnosis, treatment, and family counseling for patients. In one illustrative case report, a patient with LRBA deficiency was initially diagnosed with JIA. Treatment with NSAIDs, corticosteroids, and methotrexate was unsuccessful, and the patient developed cytopenias resistant to intravenous immunoglobulin, rituximab, cyclosporine, and splenectomy. Genetic testing identified LRBA deficiency, and the patient was started on targeted therapy with abatacept, which led to clinical improvement until she received a hematopoietic cell transplant (HCT) (159). This and other cases also illustrate that identification of an IEI can lead to not only targeted biologics but also HCT as a therapy in patients in whom it was not considered to be an appropriate therapy.

Genetic testing should be offered to patients regardless of family history, and the lack of a family history of similar disease should never preclude genetic testing. Many immune dysregulation syndromes and other IEI associated with rheumatologic disease are dominant, and oftentimes patients have de novo variants. Alternatively, some disorders have incomplete penetrance, where not all individuals carrying the disease-causing variant have clinical symptoms; for example, SOCS1 haploinsufficiency.

Given the monogenic nature of IEI, patients often present at very young ages compared to polygenic/complex autoimmune disorders. For example, SLE generally affects individuals between the ages 15–44 with a female predominance (160), whereas monogenic SLE presents in both males and females, often prior to the age of 5 years (116). Therefore, early onset autoimmunity should raise a high clinical suspicion for IEI. Other indications for genetic testing include the development of multiple immune dysregulation conditions or autoimmunity/autoinflammation refractory to standard of care. However, with increased access to genetic testing and decreasing costs, genetic testing may be considered for any patient with immune dysregulation.

There are several options for genetic testing in the clinic. Sequencing panels focus on genes known to be associated with IEI and typically are performed as exome sequencing (ES) with reporting of only the genes on the in silico panel. Clinical ES and whole-genome sequencing (WGS) are increasingly becoming available in clinical practice and, in some cases, favored by insurance companies as more comprehensive testing. WGS has the advantage of detecting intronic variants, as well as large structural variants (SVs) and copy number variants, although functional validation and interpretation of such variants can be complicated (161). While WGS is becoming more accessible in the clinic, chromosomal microarrays may still be useful in identifying large SVs, such as Chr22q11.2 microdeletion. ES and WGS have vastly improved our ability to detect genetic variants; however, there are some limitations, including difficulty detecting somatic mosaicism and resolving sequencing when there are pseudogenes, as is the case with *NCF1* and *IKBKG* (162, 163). For patients with suspected somatic mosaicism leading to disease, for example, suspected VEXAS syndrome, clinical testing may need to be targeted to the potential genes causing disease, and *UBA1* (the gene associated with VEXAS) is now on many clinical deep-sequencing panels. However, diagnostics for most mosaic disorders remain limited (164).

#### Interpretation of genetic testing

Interpreting the results of genetic testing can present multiple challenges. While some results may demonstrate a variant established to be pathogenic, frequently variants of uncertain significance (VUS) are identified, which can be difficult to interpret. It is important to consider variants within the context of the clinical presentation and evaluate whether the clinical phenotype is consistent with known phenotypes for that gene, or whether the known function of the gene could reasonably cause the observed phenotype. This is particularly relevant as different variants in the same gene may lead to distinct clinical phenotypes, for example, GOF or LOF STAT3 and multisystem immune dysregulation or hyper-IgE syndrome, respectively. Pathogenic variants causing IEI are typically rare in the population and databases such the Genome Aggregation Database are useful in identifying variant prevalence (165). Importantly, a variant may be rare among the general population but enriched in some populations, indicating that the variant is less likely to be pathogenic. Additional tools may be helpful for predicting whether a nucleotide change will alter protein function, such as CADD, AlphaFold, and others (166, 167). When possible, evaluating the genetics of both affected and unaffected family members can provide valuable information, and family testing can be helpful for resolving VUSs. However, incomplete penetrance, especially in the case of haploinsufficiency, can make interpretation of inheritance difficult (168). Finally, to determine pathogenicity, rigorous laboratory testing in a model system should evaluate whether the variant alters the function of the encoded protein in a manner consistent with disease, highlighting the importance of laboratory research in advancing the clinical diagnosis of IEI.

#### Functional testing

With genetics testing becoming more affordable and available in the clinic, a genetics-first approach is now often taken to diagnose IEI; however, functional testing still plays a vital role in the diagnosis and management of IEI, particularly with inconclusive genetics. Flow cytometry can provide insight into which cell types are affected and potentially lead to a diagnosis, for example, expanded CD4^−^CD8^−^TCRab^+^ T cells in ALPS (169). Some tests can directly assess protein function, such as the ADA2 function test (138). Evaluation of serum cytokines can also provide additional information to guide treatment plans. CXCL9 is a chemokine induced by IFN-γ, and elevated levels are associated with a wide range of immune dysregulation phenotypes and can help guide treatment decisions, for example, with JAKinibs or anti-IFNγ antibody. Patients with APECED were identified to have a strong IFNγ signature and responded to JAKinib treatment (170). For patients with suspected type I interferonopathies, clinical and research-based assays to detect transcriptional programs induced by type I IFN signaling can help to confirm a diagnosis and monitor response to therapy (171). Type 1 interferon signatures have also been identified in subsets of patients with IEI, for example, patients with HA20 with a type I IFN signature may respond to JAKinibs (172). In cases with inconclusive genetics but suspected IEI, such functional testing can help to guide treatment options.

#### Patients with “inconclusive” genetic testing

Despite advancements in sequencing technology, ∼60–70% of patients being evaluated for an IEI do not receive a genetic diagnosis (173). Current sequencing technology is best suited to detect single-nucleotide variants in coding regions of proteins; however, alternative genetic mechanisms can result in IEI. Somatic mosaicism, which occurs when a postzygotic mutation is present in only some cells, has been described in multiple IEI, for example, *FAS*, *TLR8*, and *UBA1* (174). Current sequencing technologies of ES and WGS may not sequence sufficient depth to detect low-frequency somatic variants, or the variant may not be present in the tissue sequenced (174). Additionally, SVs are difficult to detect with targeted panels and ES. WGS can detect SVs and is being more commonly used as costs decrease, but interpretation of the clinical significance of SVs is challenging (175, 176). WGS also has a trade-off of lower depth of coverage (generally ∼30x), making it difficult to detect mosaicism. Epigenetic mechanisms can also affect the penetrance of IEI. This was recently demonstrated in families heterozygous for a disease-causing variant with incomplete penetrance due to autosomal random monoallelic expression resulting in expression of either the wild-type (healthy) or mutant allele in immune cells. Such monoallelic expression can make it difficult to identify the inheritance pattern and determine pathogenicity of a variant (177). Phenocopies of IEI due to circulating autoantibodies targeting self-antigens represents another mechanism of disease that can be challenging to diagnose (178, 179). It is also likely that not all IEI are monogenic. One study found that ∼5% of cases with molecular diagnoses had multiple affected loci (180). Finally, as new diseases are discovered and variant detection algorithms continue to improve, reanalysis of existing clinical or research data can be helpful for patients with inconclusive testing.

## Treatment of rheumatologic disease in IEI

Treatment of IEI and rheumatologic conditions associated with IEI varies based on clinical symptoms and genetic cause of disease. Particularly relevant for the rheumatologist are the now vast array of biologic and other targeted therapies that can be repurposed for IEI (181). Prior to a genetic diagnosis, patients often fail multiple therapies. More than 30% of patients with STAT3 GOF were treated with five or more different therapies for their disease (95). In some cases, existing therapeutics have been repurposed to allow for precision therapy of IEI, although for the most part clinical trials are lacking to provide support for such therapies, and physicians must rely upon evidence from case reports. JAKinibs have been used with success in patients with a variety of JAK/STAT pathway GOF disorders (182, 183, 184). CTLA4-Ig fusion protein (abatacept), originally developed to treat rheumatoid arthritis, has been repurposed to treat CTLA4 haploinsufficiency and LRBA deficiency (185, 186). A recent study of 98 patients with LRBA deficiency or CTLA4 haploinsufficiency demonstrated success of abatacept, with 79% of patients exhibiting a complete clinical response (86).

One example of a drug specifically developed, tested, and now Food and Drug Administration–approved for treatment of an IEI is leniolisib, a small molecule inhibitor of PI3Kδ used to treat APDS (187). In the future, identification of additional mechanisms of immune dysregulation and dysfunction in IEI will hopefully make the development of further therapies possible.

HCT can be considered to treat patients when the defect in the hematopoietic compartment is known to cause disease, such as early onset autoimmunity in IPEX or lymphoproliferation in INFLTR8 (80, 106). There have been tremendous strides in HCT, including reduced-intensity conditioning, and while HCT is the clearly indicated for some IEIs, such as SCID, its effectiveness for other disorders, particularly those with immune dysregulation, is less certain (188). There are risks to HCT, including GVHD and, in some cases, lifelong immunosuppression (189); however, the risks of long-term immune modulation, for example, with a JAKinib to treat a JAK/STAT GOF disorder are also unknown. Finally, targeted gene therapy may soon present an alternative to HCT, including CRISPR-based techniques showing promise in treating IEI (190).

## Conclusion

IEI can manifest with a wide array of rheumatologic presentations, and some clinical signs, such as early onset, treatment-refractory conditions, and multi-organ autoimmunity, should raise a high index of suspicion that the patient may have an underlying IEI. Prompt recognition and treatment of IEI can allow for a precision diagnosis and guide therapeutic decisions. Genetic testing should be considered for any patients suspected to have a monogenic IEI leading to rheumatologic disease. With increased availability of genetic testing and an ever-growing number of known monogenic IEI, we may also be surprised to find IEI in those patients in whom there is initially low clinical concern, potentially avoiding the accumulation of autoimmunity and disease burden with early diagnosis.
